# A Practical Treatise on Nasal Catarrh and Allied Diseases

**Published:** 1885-08

**Authors:** 


**A Practical Treatise on Nasal Catarrh and Allied Diseases.** By BEVERLEY ROBINSON, A. M., M. D., Clinical Professor of Medicine in the Bellevue Hospital Medical College, &c., &c. Second edition ; revised and enlarged; with 152 wood engravings. New York: Wm. Wood & Co., 56 and 58 Lafayette Place. 1885.

In the preface to the second edition, the author states that he has added five chapters to the work as it first appears, viz.: 1. Aural complications of catarrhal inflammation of the nose. 2. Deflections of the nasal septum and bony obstructions of the nasal passages. 3. Ulcerous coryza. 4. Adenoid vegetations of the vault of the pharynx. 5. Mucous nasal polypi. With these additions in the present edition, the work is very full and complete, and is an excellent authority on the special diseases of which it treats. As such we commend it to our readers.

